# Supplementary material for: Recalibrating vision-for-action requires years after sight restoration from congenital cataracts
Source: eLife. 2022 Oct 24;11:e78734. doi: 10.7554/eLife.78734 (PMC9633067; doi:10.7554/eLife.78734)
Supplement: Figure 1—source data 1. — Participant ID, sex, age at test, pre-operation visual assessment, visual acuity (measured as contrast sensitivity function (CSF) cutoff frequency in cycles per degree (cpd)) before and after surgery, and time since surgery at test (years (y), months (m), days (d)). In the pre-surgical visual assessment, LP indicates only light perception, followed by hand motion perception (HM), and the ability to count fingers up to the specified distance (FC). We report the highest measure participants could perform with both eyes, unless otherwise specified (RE: right eye, LE: left eye). We did not test the visual acuity of four participants before surgery, either because they had too poor visual acuity to be able to perform the CSF test, or because the procedure was not available at the time they were surgically treated. Before surgery, most participants were classified as suffering from legal blindness or severe low vision. Legal blindness is defined as visual acuity below 20/400 (i.e. around 1.5 cpd cutoff frequency) by the World Health Organization (WHO 2010, International Classification of Diseases, 10th revision), or below 20/200 (i.e. 3 cpd cutoff frequency) by the National Institute of Health of the United States (NIH). Importantly, most participants improved after surgery (log-transformed pre- vs. post-surgical CSF, t15=3.7, p=0.002) and transitioned out of the category of legal blindness. We assessed the post-surgical CSF cutoff frequency in the same experimental session as the experimental task. Some participants were tested in the study multiple times, before and/or after surgery. We do not have information regarding the exact date of surgery of two participants, because they were included in our project only after surgery, and not operated by our team. Both of them were surgically treated more than 2 years before taking part in the present experiment. [file elife-78734-fig1-data1.docx]

| ID | Sex | Age (year) | Pre-op visual assessment up to | Pre-op CSF cutoff (cpd) | Post-op CSF cutoff (cpd) | Time since surgery (y,m,d) |
| --- | --- | --- | --- | --- | --- | --- |
| p1  p2  p3  p4  p5  p6  p7  p8  p9  p10  p11  p12  p13  p14  p15  p16  p17  p18  p19  p20* | m  m  f  f  f  m  m  m  m  f  m  f  m  f  m  m  f  f  f  m | 12.6  14.4  15.4  19.4  18.3  14.1  10.1  11.1  11.1  20.6  11.1  13.4  9.1  10.1  9.0  15.0  8.0  10.6  15.0  15.0 | HM  unknown  unknown  unknown  HM  FC 3m  RE: FC 1.5 m/ LE: No LP  RE: FC 50 cm/ LE: No LP  LP  FC 10 cm  HM  HM  HM  HM  RE: FC 20 cm/ LE: No LP  HM  FC 1m  FC 3 m  FC 2m  LP | unknown  unknown  unknown  unknown  0.44  3.40  1.90  1.77  0.06  0.74  1.01  0.60  0.71  2.03  0.60  1.50  1.31  2.84  2.91  0.08 | 8.24  13.04 / 13.72  3.48  2.47  12.14 / 11.99  6.73 / 7.88  13.45  2.53 / 1.49  2.99  2.88  2.30 / 1.35  2.72  3.02 / 5.37  4.05 / 5.29  1.41 / 2.24  1.58 / 1.52  6.00 / 6.19  5.82 / 9.54  4.72 / 12.74  1.30 / 1.55 | unknown  unknown/ 4m later  10.4 y  10.4 y  2.3 / 2.8 y  2.1 / 3.4 y  1.1 y  1.1 / 1.6 y  1.1 y  1.1 y  1.9 / 6.4 m  1.9 m  1.9 / 6.4 m  1.9 / 6.4 m  3 d / 4.3 m  - 2 d / 3 d / 4.3 m  -2 d / 2 d / 4.3 m  -2 d / 2 d / 4.3 m  -2 d / 1 d /4.3 m  1 d / 4.3 m |
